# Polyoxometalates in Electrochemical Energy Storage: Recent Advances and Perspectives

**DOI:** 10.3390/ijms262110267

**Published:** 2025-10-22

**Authors:** Wenjing Bao, Chao Feng, Chongze Wang, Dandan Liu, Xing Fan, Peng Liang

**Affiliations:** 1 College of Chemical and Biological Engineering, Shandong University of Science and Technology, Qingdao 266590, China; baowenjing@dicp.ac.cn (W.B.); fanxing@sdust.edu.cn (X.F.); liangpeng202@hotmail.com (P.L.); 2 Dalian Institute of Chemical Physics, Chinese Academy of Sciences, Dalian 116023, China; 3 School of Health and Life Sciences, University of Health and Rehabilitation Sciences, Qingdao 266113, China; 4 College of New Energy, China University of Petroleum (East China), Qingdao 266580, China; liudandan@upc.edu.cn

**Keywords:** polyoxometalates, electrochemical energy storage, multielectron redox, batteries and supercapacitors, functional materials

## Abstract

Polyoxometalates (POMs) are nanoscale anionic clusters constructed from transition-metal oxide units with well-defined architectures and tunable electronic structures, offering abundant reversible redox sites and adjustable energy levels. Their diverse valence states and compositional flexibility of molecular architectures render them promising candidates for electrochemical energy storage. Rational molecular design and nano-structural engineering can significantly enhance the electrical conductivity, structural stability, and ion transport kinetics of POM-based materials, thus improving device performance. In solar cells, the tunable energy levels and light-harvesting capabilities contribute to enhanced photoconversion efficiency. In secondary batteries, the dense redox centers provide additional capacity. For supercapacitors, the rapid electron transfer supports high power density storage. This review systematically summarizes recent advances in POM-based functional nanomaterials, with an emphasis on material design strategies, energy storage mechanisms, performance optimization approaches, and structure–property relationships. Fundamental structures and properties of POMs are outlined, followed by synthesis and functionalization approaches. Key challenges such as dissolution, poor conductivity, and interfacial instability are discussed, together with progress in batteries and hybrid capacitors. Finally, future challenges and development directions are outlined to inspire further advancement in POM-based energy storage materials.

## 1. Introduction

Amid the global shift toward low-carbon energy, the development of renewable-dominated power systems has become essential for achieving carbon neutrality goals [1,2,3,4]. However, the inherent intermittency and volatility of renewable electricity generation pose serious challenges to the safety and efficiency of energy storage systems [5,6]. Electrochemical energy storage, due to its operational flexibility, offers a viable solution to mitigate renewable energy fluctuations. As a critical component of such systems, electrode materials must combine molecular-level tunability with scalability for mass production [7]. Polyoxometalates (POMs), a class of molecular materials with precisely defined structures and versatile redox states, exhibit compelling advantages in meeting these requirements [8,9,10]. Composed of transition metals (such as Mo, W, and V) bridged by oxygen atoms, POMs derive their distinctive properties from several key features [11,12,13,14]. Firstly, their molecular-scale nanostructures impart unique chemical reactivity and physical properties, which can be further tuned through the introduction of organic or organometallic groups. Secondly, POM clusters undergo reversible, multi-electron transfer reactions without altering their fundamental structure, making them ideal active materials for energy storage. Furthermore, covalent networks based on coordination bonds and intermolecular interactions enable the construction of novel POM architectures, expanding their potential in functional material design.

Benefiting from these unique compositional and structural properties, POMs exhibit well-defined molecular configurations, abundant redox-active sites, and highly tunable electronic structures [15,16]. These attributes make them promising candidates for electrochemical energy storage. Rational molecular design and nanostructural control can enhance the electrical conductivity, structural stability, and ion transport kinetics of POM-based materials, thereby improving their electrochemical performance. The structural and compositional diversity of POMs-incorporating multiple redox-active metal centers (e.g., Mo^5+/6+^, W^5+/6+^, and V^4+/5+^) allows for precise tuning of redox potentials at the molecular or even atomic level [17]. Their exceptional multi-electron storage capability, often termed “electron sponge” behavior, enables additional capacity through reversible multi-electron transfers [12,18]. Owing to these advantages, POM-based functional nanomaterials show broad application prospects in various energy storage devices, including solar cells [19,20,21], lithium-ion batteries [22,23,24,25], lithium–sulfur batteries [26,27], sodium-ion batteries [28,29,30], and supercapacitors [31,32,33,34].

**Figure 1:**
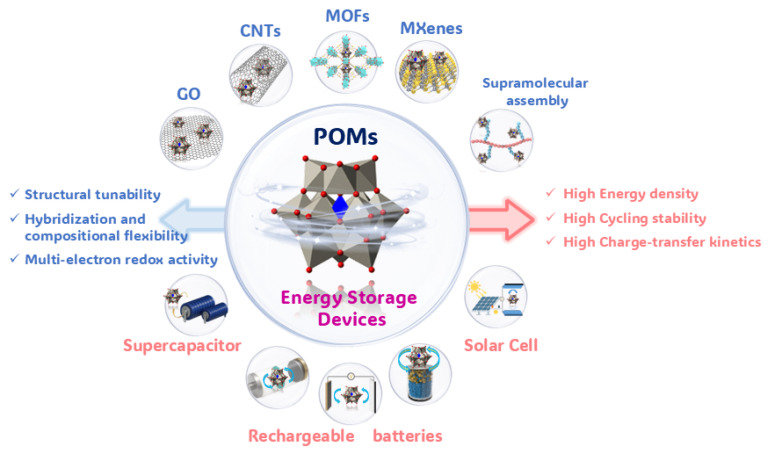
Overview of POM-based materials for electrochemical energy storage.

Although previous reviews have summarized fundamental progress in POM-based materials for energy storage, systematic analysis of their commercialization challenges and performance under practical operating conditions remains limited. This review evaluates the industrial potential of POM-based materials from a scalable application perspective, focusing on synthetic scalability, cost-effectiveness, and long-term stability under realistic operational environments. We first outline precise design strategies and research frontiers in functional POMs and then categorize key scientific and technical challenges in developing high-energy-density, high-power-density, and long-lifespan energy storage systems, highlighting the unique role of POMs in overcoming current energy storage limitations. By comprehensively analyzing their performance in enhancing system-level energy/power density and cycling stability, we summarize critical advances toward practical implementation, particularly in understanding structure–property relationships and charge storage mechanisms. Finally, we identify promising research directions and development pathways to address engineering challenges in translating POM materials from laboratories to real applications (Figure 1).

## 2. Overview of the Properties for POMs

**Figure 2:**
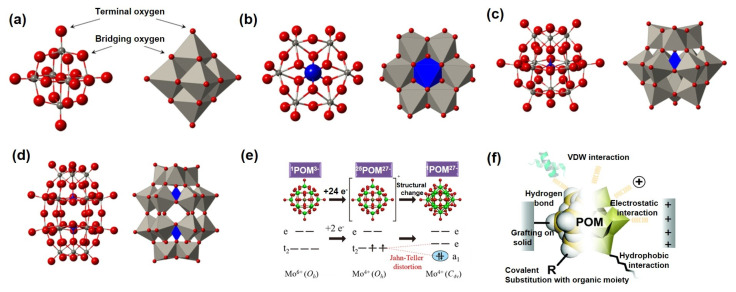
(**a**) Structural formula of POMs; (**b**–**d**) structural formulas of Anderson-type, Keggin-type, and Dawson-type POMs. Reprinted with permission from Ref. [37], Copyright 2018, *Chemical Reviews*. (**e**) Multi-electron transfer properties of POMs. Reprinted with permission from Ref. [46], Copyright 2012, *Journal of the American Chemical Society*. (**f**) Structural characteristics of POMs. Reprinted with permission from Ref. [18], Copyright 2018, *Chemical Society Reviews*.

Polyoxometalates (POMs) exhibit versatile architectures, among which the Keggin-, Dawson-, and Anderson-type structures are most prevalent. These nanoscale molecular clusters, adorned with abundant surface oxygen atoms, offer an ideal platform for functionalization and advanced material design (Figure 2a) [35,36,37].

The Anderson-type POMs, with the general formula [XM_6_O_24_]^n−^ (X = heteroatom, e.g., Te^6+^, Ni^2+^; M = Mo^6+^, W^6+^), exhibit a planar hexagonal framework of six edge-sharing MO_6_ octahedra centered on a heteroatom (Figure 2b). This unique configuration enables [38,39,40]: (i) isotropic electron distribution due to high symmetry; (ii) exposed surface-active sites for electron transfer and ion adsorption; and (iii) electronic tunability via heteroatom substitution. Their stability in protonation/deprotonation supports pH-sensitive electrocatalysis and energy storage applications, while surface functionalization or heteroatom engineering further enhances their versatility. The Keggin-type POMs, represented as [XM_12_O_40_]^n−^ (X = P^5+^, Si^4+^; M = Mo^6+^, W^6+^), feature a central XO_4_ tetrahedron enclosed by 12 MO_6_ octahedra in a symmetric cage (Figure 2c). This architecture delivers [41,42,43]: (i) exceptional chemical and thermal stability; (ii) uniform distribution of surface oxygen atoms for diverse coordination; and (iii) tunable electronic band structures via core-shell coupling. Their ability to maintain structural integrity during redox processes makes them ideal for applications in supercapacitors and batteries. Dawson-type POMs, with the formula [X_2_M_18_O_62_]^n−^, can be viewed as two Keggin units linked by shared oxygen atoms (Figure 2d). This dimeric structure provides [12,44,45]: (i) augmented redox-active sites; (ii) a hollow cage suitable for guest-encapsulating cavity; and (iii) inter-subunit electronic synergy. Notably, Dawson-type POMs surpass Keggin analogues in electron storage capacity and structural diversity, permitting precise modulation of redox potentials and acid–base properties through atomic substitution, thus excelling in multi-electron catalysis.

A defining electrochemical feature of POMs is their exceptional multi-electron redox capability, originating from the variable valence states of transition metals. For instance, Keggin-type H_3_[PMo_12_O_40_] can reversibly accept up to 24 electrons without structural degradation (Figure 2e), endowing it with high theoretical capacity [46]. The redox potentials of POMs can be systematically tuned by altering the heteroatom, metal atom, or counterion (e.g., H^+^ and NH_4_^+^), enabling the design of electrode materials with tailored operating voltages [47,48].

Additionally, solid-state POMs exhibit distinctive pseudo-liquid behavior: their dynamic metal–oxygen networks enable rapid ion diffusion and reversible insertion/extraction akin to liquid-phase systems, despite maintaining structural solidity [49]. This property enhances rate capability while suppressing volume strain during cycling, significantly improving electrode durability [18,50]. Moreover, the abundant bridge and terminal oxygen atoms on POM surfaces further facilitate strong interactions with carbon materials or conductive polymers via coordination, hydrogen bonding, or electrostatic forces, enabling the construction of high-performance composite electrodes for stable energy storage systems.

In summary, POMs possess several distinctive attributes (Figure 2f) [18]. (i) molecular-scale nanostructures with tunable reactivity and physical properties; (ii) reversible multi-electron transfer without structural collapse, ideal for energy storage; and (iii) capacity to form covalent networks through coordination and intermolecular interactions, expanding their applicability in functional material design. Their structural versatility, multi-electron redox activity, and molecular flexibility offer significant opportunities for developing next-generation electrochemical energy storage systems.

## 3. Overview of POM-Based Functionalized Materials

### 3.1. POMs/Nanocarbon Composites

**Figure 3:**
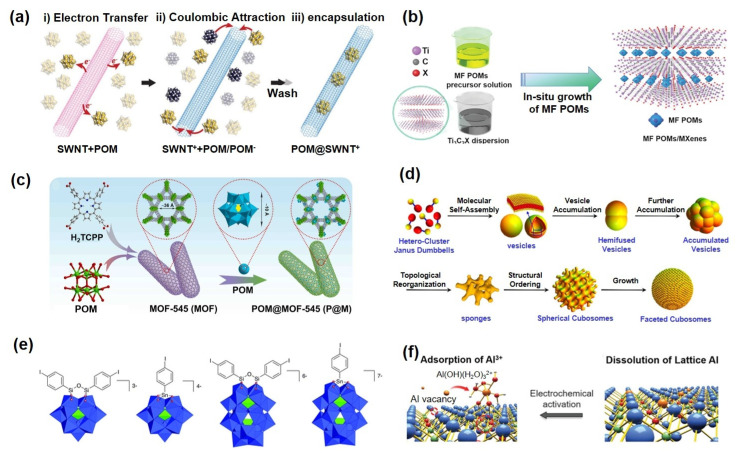
(**a**) Schematic illustration of POMs encapsulated in CNTs. Reprinted with permission from Ref. [54], Copyright 2019, *Advanced Materials*. (**b**) Schematic illustration of POM/MXene composite materials. Reprinted with permission from Ref. [58], Copyright 2021, *Advanced Functional Materials*. (**c**) POMOF complex structure. Reprinted with permission from Ref. [62], Copyright 2022, *Nano Energy*. (**d**) Schematic diagram of the self-assembly of POM molecules. Reprinted with permission from Ref. [71], Copyright 2019, *Journal of the American Chemical Society*. (**e**) POM-based inorganic–organic hybrid materials. Reprinted with permission from Ref. [17], Copyright 2012, *Chemical Society Reviews*. (**f**) SACs anchored by POMs. Reprinted with permission from Ref. [76], Copyright 2022, *Advanced Science*.

Recent advances in POM-carbon composites demonstrate significant potential for boosting electrochemical performance. Carbon nanotubes (CNTs) provide unique nanoscale cavities that serve both as confined reactors and conductive supports for POM-based materials. Encapsulation of active catalytic components within CNTs enables precise size control of POM nanoparticles tailored to the tube diameter. More importantly, the spatial confinement within the cavity modulates the electronic properties of the encapsulated POMs, significantly enhancing their catalytic activity. Awaga et al. [51] pioneered the integration of Keggin-type POMs (TBA_3_[PMo_12_O_40_]) with single-walled carbon nanotubes (SWNTs), demonstrating that POMs can be stabilized via chemical adsorption on SWNT surfaces, forming composite electrodes with rapid electron transfer and Li^+^ diffusion capabilities. Compared to crystalline POMs, these amorphous composites exhibited higher lithium storage capacity and faster charge–discharge kinetics. However, the weak interaction between POMs and CNTs often leads to material detachment during cycling. To address this, organic functionalization strategies have been developed to strengthen POM-CNT interactions. Song et al. [52] constructed a Py-Anderson-CNT composite via π-π stacking, which significantly improved rate capability and cycling stability. Nonetheless, limited modification sites and potential disruption of CNT electronic structure remain challenges. The same group subsequently developed an ultrasound-assisted deposition method to achieve controlled assembly of POM nanocrystals on CNTs, yielding electrodes that maintained a high capacity of 850 mAh·g^−1^ after 100 cycles [53]. Khlobystov et al. [54] employed host–guest self-assembly to fabricate POM@SWNT hybrids, revealing that SWNTs not only act as electron donors to stabilize POMs but also mitigate their chemical degradation. Research has expanded to other carbon substrates (Figure 3a). Graphene oxide (GO) and reduced graphene oxide (RGO), with their high surface area and conductivity, serve as excellent supports. Yoshikawa et al. [24] reported that POM/RGO composites combine high energy and power density, attributed to synergistic effects between the rapid ion transport in RGO and the multi-electron redox capability of POMs. Lan et al. [55] introduced ionic liquids (ILs) as structural directors to uniformly assemble POM nanobelts on RGO surfaces. Recent focus has turned to two-dimensional transition metal carbides/nitrides (MXenes). Despite their high conductivity and tunable surface chemistry, MXene-based electrodes suffer from low initial Coulombic efficiency and interlayer restacking [56,57]. Cao et al. [58] innovatively anchored POMs onto MXene surfaces via chemical bonding, leveraging synergistic effects to achieve high-loading and efficient pseudocapacitive energy storage (Figure 3b). This work provides a strategic approach toward developing stable, high-capacity energy storage materials, highlighting the broad potential of POM-carbon hybrid systems in electrochemical energy applications.

### 3.2. POMOF Composites

Strategically integrating the molecular-scale dispersibility and reversible multi-electron redox capability of POMs with the tunable pore architectures, unique cavity environments, and surface chemistry of metal-organic frameworks (MOFs) offers a promising route to high-performance energy storage devices [59]. In recent years, POM-based MOFs (POMOFs) have garnered significant attention as emerging electrode materials (Figure 3c), exhibiting the following key features in electrochemical energy storage [60,61,62]. (i) the inherent redox activity of POM clusters provides uniformly distributed and accessible active sites within the framework; (ii) POMOFs enable precise modulation of the physicochemical properties of electrodes at the molecular level, enhancing functionality through multi-component synergy; and (iii) confinement of POMs within MOF channels effectively prevents aggregation and improves both dispersion and electrochemical stability of the active species. Lan et al. [63] synthesized a novel [PMo_8_VMo_4_VIO_37_(OH)_3_Zn_4_][TPT]_5_·2TPT·2H_2_O (NNU-11) composite via solvothermal assembly. This material features a two-dimensional network constructed from Zn-ε-Keggin POM units connected with organic ligands via coordination bonds, which further stack into a three-dimensional porous framework through π-π interactions. This architecture offers dual advantages: the extended π-conjugated system facilitates efficient electron transport, while the multi-electron redox activity of POMs synergizes with lithium storage sites on organic ligands, significantly enhancing lithium storage capacity. Electrochemical tests revealed a reversible capacity of 750 mAh·g^−1^ after 200 cycles at 50 mA·g^−1^, demonstrating exceptional cycling stability. To address the limited intrinsic conductivity of POMOFs, Jiang et al. [64] developed a ternary composite material, denoted as [Cu_12_(Trz)_8_Cl][PMo_12_O_42_V_2_]@PPy/RGO, which integrates a vanadium-substituted Keggin-type polyoxometalate cluster, a copper-organic framework constructed from triazole ligands, and a conductive matrix comprising polypyrrole (PPy) and RGO. This design incorporated several key strategies: a vanadium-substituted Keggin-type POM with high redox potential was employed as the active center; a triazole-based MOF with size-matched pores confined the POM clusters; and in situ polymerized PPy served as a conductive binder, while RGO provided a three-dimensional conductive network. This hierarchical structure enhanced interfacial adhesion and electronic conductivity within the electrode. The composite delivered a high reversible capacity of 985 mAh·g^−1^ after 100 cycles at 50 mA·g^−1^, along with remarkable rate capability, offering a new design paradigm for high-performance POMOF-based electrodes.

### 3.3. Molecular- and Atomic-Level Self-Assembly of POMs

The development of advanced energy storage electrode materials faces a central challenge: achieving efficient ion and charge transport through rational structural design and interface engineering. Self-assembly techniques enabling the construction of multi-level ordered architectures offer a promising route for precise control over the physical and chemical interfaces of electrodes. The performance of such materials critically depends on the spatial precision of component arrangement and the strength of intermolecular interactions [65,66,67]. However, conventional molecular building blocks often suffer from structural simplicity, limited binding sites, and weak driving forces for assembly, which restrict further tuning of material properties. In this context, POMs have emerged as ideal molecular platforms due to their well-defined structures, sub-nanometer dimensions, rich coordination environments, and chemical diversity, allowing accurate design of both bulk and surface properties of electrode materials (Figure 3d) [68,69,70,71]. Achieving molecular-level long-range ordered assembly of POMs typically requires synergistic use of electrostatic interactions with cationic species, supplemented by auxiliary driving forces such as chaotropic effects, void filling, or nanophase separation [16], leading to the formation of structurally diverse and functionally tunable inorganic materials with significant potential for next-generation energy storage technologies (Figure 3e) [17].

Moreover, single-atom catalysts (SACs) have attracted considerable attention in energy storage owing to their unique low-coordination metal centers, nearly maximum atom utilization efficiency, and tunable metal-support interactions [72,73,74]. Each metal atom in SACs can serve as an active site that dynamically couples with its local environment, exhibiting exceptional catalytic activity [73,75]. However, their high surface energy and tendencies toward leaching and aggregation under operational conditions have impeded practical application. POMs, with their well-defined molecular structures and tunable electronic properties, provide ideal anchoring sites for stabilizing and hosting single atoms (Figure 3f) [76]. Two main strategies are commonly employed: (i) the use of lacunary POM anions with high negative charge to isolate and stabilize metal atoms within specific framework sites, significantly enhancing the stability and reactive diversity of metal centers [77,78]; and (ii) the application of plenary POM clusters as substrates where metal atoms are adsorbed via surface low-coordination oxygen sites-a method that allows tunable loading but generally offers moderate stability [79]. Both approaches yield metal centers in an unsaturated coordination state, resulting in high reactivity. It is important to note that the anchoring environment not only influences the redox properties of POMs but also considerably modulates the chemical behavior of supported SACs [80]. Integrating the well-defined molecular model of POMs with the design concept of SACs holds great promise for advancing energy storage materials toward atomic-level precision and functional integration.

## 4. POM-Based Materials for Energy Storage Device

### 4.1. Solar Cell

**Figure 4:**
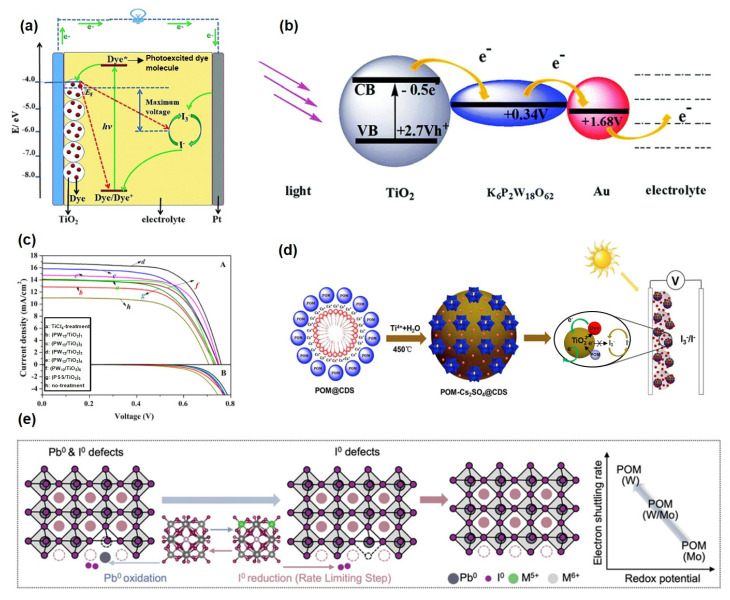
(**a**) The working principle of DSSCs. Reprinted with permission from Ref. [84], Copyright 2019, *Chemical Society Reviews*. (**b**) Multi-electron transfer mechanism of POMs as photosensitizers. Reprinted with permission from Ref. [100], Copyright 2012, *Journal of Materials Chemistry*. (**c**) Current−voltage curves (A) and dark current curves (B). Reprinted with permission from Ref. [94], Copyright 2014, *Industrial & Engineering Chemistry Research*. (**d**) Schematic illustration of the functional mechanisms of POMs in DSSCs. Reprinted with permission from Ref. [98], Copyright 2016, *Journal of Power Sources*. (**e**) Harnessing POMs’ redox properties for enhanced PSC performance. Reprinted with permission from Ref. [88], Copyright 2024, *Advanced Materials*.

Solar energy, recognized as one of the most promising renewable energy sources, is increasingly considered a viable alternative to conventional fossil fuels due to its cleanliness, sustainability, and wide distribution. Among solar energy conversion technologies, dye-sensitized solar cells (DSSCs) have attracted significant research interest owing to their simple fabrication, low cost, and environmental friendliness [81,82,83]. As shown in Figure 4a, the operational mechanism of DSSCs involves several key steps: photosensitive dyes absorb photons, exciting electrons from the ground state to a higher energy level [84]. These excited electrons are subsequently injected into the conduction band (CB) of a semiconductor (e.g., TiO_2_), leading to charge separation. Electrons in the CB are then transported through the semiconductor to the external circuit, while the oxidized dye molecules are regenerated to their original state by reductants in the electrolyte, thereby completing the photoelectric conversion cycle. However, the power conversion efficiency of DSSCs remains limited by intrinsic challenges such as severe charge recombination and narrow light-harvesting ranges [85,86]. Recently, POMs have emerged as promising functional materials in DSSCs due to their unique electronic structures, tunable energy levels, and exceptional photoelectrochemical properties [84,87,88,89]. Based on their distinct functional roles in DSSCs, POMs can be systematically categorized into the following types: photosensitizers or electron acceptor materials [90,91], electrolytes or electrolyte additives [92,93], interfacial/electron transport layers [94,95,96], and photoanode modifiers [97,98,99].

#### 4.1.1. Photosensitizers or Electron Acceptors

By incorporating Dawson-type POMs (P_2_W_18_) together with gold nanoparticles into a TiO_2_ matrix, Xu et al. [100] significantly enhanced the photoelectrochemical performance of the material (Figure 4b). The redox potential of P_2_W_18_ (+0.34 V) aligns well with the energy level of TiO_2_ (−0.50 V), facilitating efficient electron transfer from TiO_2_ to P_2_W_18_ and further migration to the Au nanoparticles. The synergistic effect between POMs as electron mediators and Au nanoparticles as conductive junctions effectively suppresses electron recombination and promotes the extraction of interfacial electrons to the external circuit. As a result, assembled DSSC exhibits the highest photocurrent response and power conversion efficiency (PCE). Moreover, Hugo Cruz et al. [91] developed four types of polyoxometalate-based ionic liquids (POM-ILs) incorporating phosphomolybdate anions. Electrodeposited [BPy]_3_[PMo_12_O_40_] and [P_6,6,6,14_]_3_[PMo_12_O_40_] significantly suppressed electron recombination via an “electron trap” mechanism and reduced aggregation, leading to a 37-fold efficiency improvement compared to commercial H_3_PMo_12_O_40_.

#### 4.1.2. Electrolytes or Additives

Fielden et al. [93] expanded this approach by incorporating [NBu_4_]_2_[Mo_6_O_19_] and its derivatives into p-DSSCs, leading to notable improvements in both open-circuit voltage and current output. Subsequently, Bakker et al. [92] demonstrated that Lindqvist-type POMs, specifically [TBA]_2_[Mo_6_O_19_], serve as effective redox mediators in p-type DSSCs. Their significantly lower reduction potential compared to the conventional I_3_^−^/I^−^ electrolyte considerably enhances the open-circuit voltage (reaching 423 mV for Mo-based and 541 mV for W-based systems). The multi-electron transfer capability and molecular size effects of POMs further facilitate charge transport kinetics and promote the formation of an ordered double layer at the electrode/electrolyte interface.

#### 4.1.3. Interfacial/Electron Transport Layers

Wang et al. [94] constructed a (PW_12_/TiO_2_)_3_ composite interlayer via layer-by-layer self-assembly, which prolonged electron lifetime to 30 ms and reduced dark current density by 75%, resulting in a 54% enhancement in device efficiency (Figure 4c). Gu et al. [95] modified TiO_2_ with Sm-substituted POMs (Sm-POMs), which reduced charge transfer resistance by 32% and improved the incident photon-to-current conversion efficiency (IPCE) to 1.8 times that of pure TiO_2_ in the 400–650 nm range, owing to synergistic electron-accepting and ferroelectric effects. Zhang et al. [101] functionalized CoS_2_ counter electrodes with PW_12_, which reduced the surface work function by 0.35 eV and increased the efficiency of quantum dot-sensitized solar cells (QDSSCs) from 3.75% to 6.29%, while extending electron lifetime to 4.46 ms.

#### 4.1.4. Photoanode Modifiers

In 2016, Wang et al. [98] developed highly dispersed POMs-Cs_2_SO_4_@TiO_2_ nanomaterials, which increased power conversion efficiency by ∼40% compared to pure TiO_2_ and extended the spectral response into the visible region (Figure 4d). Moloto et al. [102] employed a Bi-modified phosphotungstate coupled with FeBTC and N-CQDs to fabricate a hybrid photoanode, achieving an efficiency of 1.92% in natural dye-based DSSCs with an extended electron lifetime of 30 ms. To enhance the structural stability of perovskite solar cells (PSCs), redox-active POMs have been incorporated into the perovskite layer, forming a stable POMs/perovskite composite structure [88]. This approach offers several advantages: POMs facilitate the formation of an interfacial connection layer through ion exchange with ammonium cations in the perovskite, effectively passivating defects and stabilizing the [PbI_6_]^4−^ framework. The redox properties of POM-based electroactive metal ions enable dynamic repair of Pb^0^ and I^0^ defects, thereby maintaining structural integrity (Figure 4e).

The application of POMs as charge-transport layers or interfacial modifiers in solar cells has shown promising potential, yet their commercialization faces challenges in scalability, cost, and long-term stability. Laboratory-scale synthesis typically involves complex procedures and high-purity precursors, making it difficult to scale up to industrial production. Moreover, the separation and purification of POMs are often inefficient and energy-intensive, further limiting large-scale manufacturing feasibility. Although POMs are composed of earth-abundant elements such as tungsten and molybdenum, the cost of high-purity metal precursors and overall processing remains high compared to commercialized metal oxides like TiO_2_, undermining their economic competitiveness. Stability issues also pose critical concerns: the inherent acidity of POMs may induce interfacial reactions with adjacent layers, particularly pH-sensitive perovskite absorbers or metal electrodes, leading to material degradation and performance loss. Under real-world operating conditions, environmental stressors such as humidity, thermal cycling, and UV irradiation can further accelerate structural phase transitions or decomposition of POMs, hastening device aging. Therefore, advancing the commercial viability of POM-based solar cells requires the development of scalable and low-cost synthesis routes, design of chemically and structurally robust POM derivatives, and systematic evaluation of their long-term durability under realistic operating conditions.

### 4.2. Supercapacitor

**Figure 5:**
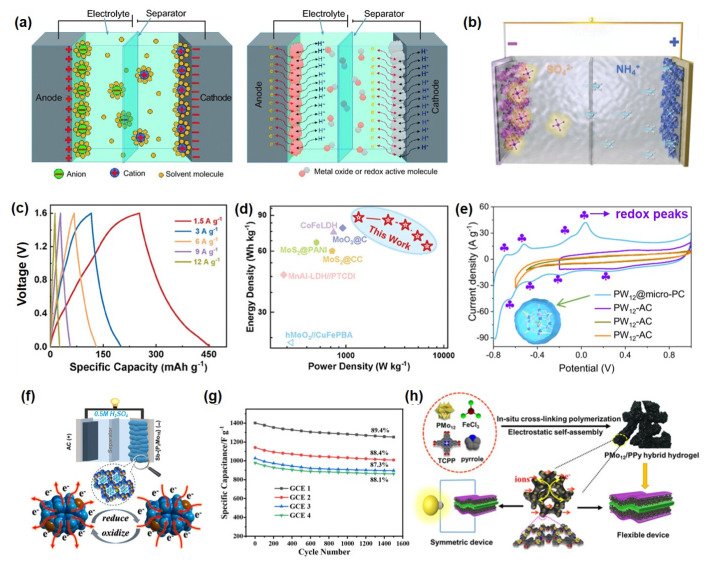
(**a**) Schematic illustration of the working mechanism of a supercapacitor. Reprinted with permission from Ref. [111], Copyright 2017, *National Science Review*. (**b**) Schematic illustration of POMs as pseudocapacitive electrodes. (**c**) GCD curves and (**d**) Ragone plot of supercapacitors with Keggin-type POM-modified Ag−BTC. Reprinted with permission from Ref. [110], Copyright 2024, *Advanced Materials*. (**e**) CV curves of Keggin−type PMo_12_. Reprinted with permission from Ref. [112], Copyright 2023, *Energy & Environmental Science*. (**f**) Working mechanism diagram of Dawson-type POM derivatives. (**g**) Cycling performance of an asymmetric supercapacitor using Dawson−type POM derivatives as electrodes. Reprinted with permission from Ref. [115], Copyright 2023, *Electrochimica Acta*. (**h**) Functional mechanism of a POM-based hydrogel electrode. Reprinted with permission from Ref. [119], Copyright 2020, *Electrochimica Acta*.

Supercapacitors represent a class of energy storage devices that bridge the gap between conventional capacitors and batteries, offering high power density (≥10 kW/kg), long cycle life, and rapid energy storage/release capabilities [103,104,105]. Energy storage mechanisms primarily include electric double-layer capacitance (EDLC), based on ion adsorption at the electrode-electrolyte interface, and pseudocapacitance, which relies on fast and reversible surface or near-surface Faradaic reactions [106,107,108]. EDLC stores charge via electrostatic accumulation in the double layer, while pseudocapacitance involves redox reactions or electrosorption in electrode materials (Figure 5a) [109,110,111]. POMs have emerged as promising pseudocapacitive electrode materials due to their rich redox activity, structural tunability, and molecular-scale controllability. POMs facilitate multi-electron transfer processes, significantly enhancing pseudocapacitive performance [112,113]. Moreover, their molecular design can be tailored to improve compatibility with electrode substrates and ion transport kinetics, leading to superior capacitive performance and cycling stability. Based on the primary functions of POMs in different components, their applications can be categorized into the following types: electrode active material [64,110,112,114,115], synergistic component in composite electrodes [116], and electrolyte functional additive or hydrogel electrode [117,118,119].

In POM-based supercapacitors, pseudocapacitance and electric double-layer capacitance operate synergistically, yet their mechanisms and contributions are distinct. Pseudocapacitance constitutes the core mechanism underpinning the exceptional electrochemical performance of POMs. It originates from the rapid and reversible Faradaic redox reactions inherent to POM molecules. Metal ions (e.g., W, Mo, and V) within the anionic POM framework undergo multi-electron transfer at their surface or throughout their bulk, accompanied by the synchronous intercalation and deintercalation of electrolyte cations (e.g., H^+^ and Li^+^) to maintain charge neutrality. This process enables POMs to function as efficient “electron sponges,” storing and releasing substantial charge via valence changes, thereby delivering specific capacitance and energy density far surpassing those of conventional carbon materials. In contrast, the electric double-layer capacitance plays a complementary yet crucial role. Its mechanism primarily manifests in two aspects: first, when POM nanoclusters are highly dispersed on high-surface-area conductive substrates, they augment the electrode’s effective surface area, providing additional sites for electrostatic ion adsorption. Second, the highly negative charges inherent to POM anions allow them to act as multi-charge “ion traps,” electrostatically concentrating electrolyte cations within their surrounding Helmholtz layer. This process is characterized by extremely fast kinetics and high reversibility, forming the basis for the device’s high power density and excellent cycling stability.

#### 4.2.1. Electrode Active Material

POMs serve directly as pseudocapacitive active materials, significantly enhancing the specific capacity and energy density of electrodes through multi-electron transfer reactions. For example, Liang et al. [110] fabricated a PMo_12_@Ag-BTC electrode material via a self-assembly strategy that integrated the carboxyl groups of Ag-BTC with terminal oxygen-enriched Keggin-type POM clusters. This approach enabled uniform dispersion of POMs on the support, effectively suppressing aggregation and exposing abundant molybdenum-oxo (Mo-O_x_) active sites at the NH_4_^+^ intercalation/deintercalation interface (Figure 5b). The PMo_12_@Ag-BTC electrode delivered a specific capacity of 619.4 mAh·g^−1^ at 1 A·g^−1^ (Figure 5c) and exhibited no capacity decay after 20,000 high-rate charge-discharge cycles. A supercapacitor assembled with PMo_12_@Ag-BTC and PMo_12_@Ag-BTC as the positive and negative electrodes, respectively, achieved a high energy density of 125.3 Wh·kg^−1^ (Figure 5d). This outstanding performance originates from the pronounced pseudocapacitance contribution of POMs owing to their excellent redox activity, coupled with the reversible NH_4_^+^ intercalation/deintercalation at the interface, which mitigates structural strain and significantly improves cycling stability. Moreover, Yang et al. [112] confined Keggin-type PMo_12_ within microporous carbon, fully activating its multi-electron redox activity (as evidenced by the distinct redox peaks of POMs observed in the CV curves, as shown in Figure 5e nd achieved an areal capacitance of 443 mF·cm^−2^ at an ultralow loading (~11.4 wt%). Jiang et al. [64] constructed a POMOF@PPy/RGO ternary composite that utilized Mo/V multi-redox centers to deliver a pseudocapacitive contribution of 56.66% at 0.2 mV s^−1^ and a reversible capacity of 985 mAh g^−1^. POMs optimize the electronic structure and interfacial properties of electrode materials through surface modification, chemical bonding, or spatial confinement. Li et al. [114] utilized W=O bonds in H_3_PW_12_O_40_ to modulate the work function of CoS_2_ (from 4.8 eV to 4.2 eV), increasing the specific capacitance to 412 F g^−1^ with 92% capacity retention after 5000 cycles. Alshehri et al. covalently immobilized Lindqvist-type POMs onto a polypyrrole framework, achieving a Mo content of 5–6% and 95% capacity retention after 1200 cycles. Zhou et al. [115] synthesized antimony-capped Dawson-type POM derivatives via a hydrothermal method. The material features a 3D supramolecular channel structure and exhibits notable pseudocapacitive behavior with multi-electron transfer (Figure 5f. An asymmetric supercapacitor based on this material achieved 89.14% capacity retention over prolonged cycling, demonstrating excellent energy storage stability Figure 5g). To address the dissolution and aggregation issues of POMs in electrolytes, structural encapsulation and composite design have emerged as pivotal research strategies. Encapsulating POM derivatives within carbon nanofibers via electrospinning produces self-supporting films (POMs-D@CNFs), which enhance ion transport kinetics and mitigate volume expansion in sodium-ion storage, significantly improving rate capability and cycling longevity [120]. Using ZIF-67 as a template to confine PMo_12_, followed by etching and selenization, yields hollow-structured MoSe_2_/(Ni,Co)Se_2_ composites; the optimized electrode maintains 83.7% capacity after 10,000 cycles, and the assembled supercapacitor delivers an energy density of 40.3 Wh·kg^−1^ [121]. Employing POMs as precursors to construct heterostructures also proves highly effective: for instance, carbon-nitrogen-doped NiMoO_4_·MoO_2_/NC materials leverage multiple redox sites and high ionic conductivity to achieve a specific capacitance of 364.96 F g^−1^ and an energy density of 102 Wh·kg^−1^ in zinc-based capacitors [122]. Furthermore, incorporating carbon dots to form CDs@PMo_12_/Ni-MOF composites elevates the specific capacity to 748.8 C g^−1^ through interfacial synergy, while demonstrating exceptional structural and electrochemical stability with 98.4% capacity retention over 20,000 cycles [123].

#### 4.2.2. Synergistic Component in Composite Electrodes

POMs are integrated with carbon materials, MXene, or polymers to synergistically enhance electrical conductivity, structural stability, and reaction kinetics. For example, an MXene-PIL-POM hybrid electrode delivers a specific capacitance of 384.6 F·g^−1^ at 1 A·g^−1^-three times that of pure MXene-while retaining 91.7% capacitance after 2000 cycles [116]. A three-dimensional PMo_12_/PPy hydrogel forms continuous ion/electron transport pathways through its cross-linked structure, achieving a specific capacitance of 776 F·g^−1^ and an energy density of 50.66 Wh·kg^−1^ in the solid-state device, along with remarkable flexibility and bending stability. Mu et al. [124] developed a polyoxometalate–amino acid supramolecular conductive gel as a flexible electrode for constructing two-dimensional (2D) supercapacitors. The gel exhibited strong interfacial adhesion with conventional PVA gel electrolytes and good compatibility with various flexible substrates, allowing direct injection or printing of the electrodes onto substrate surfaces without complex equipment. Furthermore, integrated devices printed with this gel achieved a series-connected configuration delivering over 3 V, maintaining 92% capacitance retention under repeated bending and twisting. These results highlight the feasibility of employing POM-based supramolecular conductive gels as flexible electrodes for next-generation 2D supercapacitors.

#### 4.2.3. Electrolyte Functional Additive/Hydrogel Electrode

POMs and their derivatives introduce additional redox reactions into electrolytes, broadening the operating voltage window and enhancing overall device performance. Hor et al. [117] synergistically employed TMSI with POM-based ionic liquids to trigger reversible I^−^/I_3_^−^ reactions in a gel electrolyte, enabling a specific capacitance of 613 F·g^−1^ and an energy density of 69 Wh·kg^−1^ within a 2.9 V window. Hibino et al. [118] utilized a POM-derived proton conductor, SnAlHP_2_O_7_-PTFE, which maintained a proton conductivity of 0.02 S·cm^−1^ at 200 °C, while the electrode delivered a specific capacitance of 210 F·g^−1^ and an energy density of 32 Wh·kg^−1^. Moreover, Wang et al. [119] fabricated a three-dimensional interconnected phosphomolybdic acid/polypyrrole (PMo_12_/PPy) composite hydrogel electrode via a one-step in situ crosslinking polymerization strategy (Figure 5h). Density functional theory calculations reveal that the stable hydrogen-bonding network between the crosslinker TCPP and PPy chains offers abundant adsorption sites for PMo_12_, significantly enhancing the structural stability. The assembled liquid device exhibits a specific capacitance of 300 F·g^−1^, excellent rate capability, cycling stability, and retains high capacitive performance even under bending conditions.

POMs show promise as high-performance electrode materials or electrolyte additives for supercapacitors, yet their commercialization faces three fundamental challenges. Scalable synthesis remains difficult, as the multi-step reactions and precise purification required for structurally defined POMs hinder cost-effective industrial production. Although POMs consist of earth-abundant elements like molybdenum and tungsten, their overall cost is elevated by expensive high-purity precursors and energy-intensive processing, making them less competitive than conventional activated carbon. Most critically, long-term stability is compromised by POM dissolution in electrolytes—leading to active material loss—and irreversible structural changes during continuous electrochemical cycling or under extreme temperature fluctuations, resulting in capacitance fade and device failure. Addressing these barriers requires developing scalable immobilization strategies, designing dissolution-resistant POM derivatives, and rigorously validating their durability under realistic operating conditions.

### 4.3. Rechargeable Batteries

#### 4.3.1. Lithium-Ion/Sodium-Ion Batteries

In recent years, lithium-ion batteries (LIBs) have achieved remarkable success due to their high operating voltage (>3.5 V) and high energy density [35,125]. However, their electrode materials often undergo structural transformations during repeated Li^+^ insertion/extraction, leading to capacity decay and shortened lifespan. In sodium-ion batteries (SIBs), the larger ionic radius of Na^+^ results in higher Coulombic barriers and slower diffusion kinetics, exacerbating issues such as volume expansion, phase transitions, and irreversible reactions, which severely limit reversible capacity retention [126,127]. POMs show great promise as electrode materials for both LIBs and SIBs owing to their multi-electron redox capability, tunable oxidation states, and discrete polymorphic structures. POMs enable efficient simultaneous ion and electron transfer and can serve as independent redox centers that deliver high capacity without inducing crystallographic changes. Furthermore, nanostructured materials derived from POM precursors demonstrate unique advantages in suppressing electrode phase transitions and capacity fading. The metal- and oxygen-rich nature of POM clusters provides an ideal platform for constructing porous metal nanocomposites, effectively mitigating particle aggregation and volume expansion during cycling while facilitating rapid charge transfer. By integrating POMs with organic molecules, polymers, or carbon matrices, electrode architectures can be further optimized, significantly enhancing the cycling stability and reversible capacity of metal-based electrodes.

**Figure 6:**
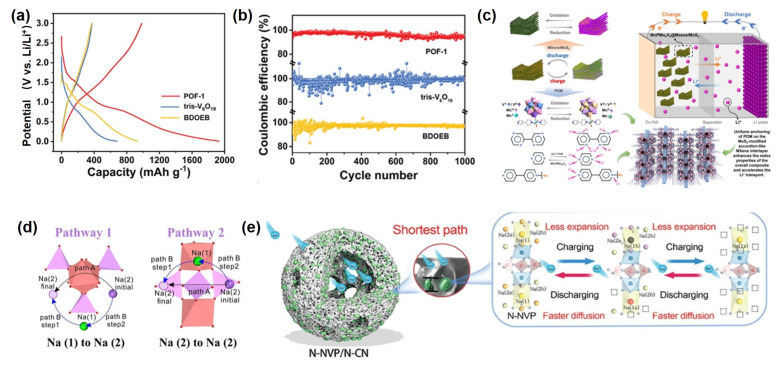
(**a**) GCD curve of POMOFs material. (**b**) Comparison of the specific capacity of POM−based and vanadium−based materials. Reprinted with permission from Ref. [130], Copyright 2023, *Advanced Functional Materials*. (**c**) Reprinted with permission from Ref. [131], Copyright 2025, *Chemical Engineering Journal*. (**d**) Diffusion path of Na^+^ in the lattice of NVP. Reprinted with permission from Ref. [132], Copyright 2018, *Journal of Physical Chemistry* C. (**e**) Sodium storage mechanism of surface−nitrogen−doped POM materials. Reprinted with permission from Ref. [133], Copyright 2024, *Advanced Science*.

In recent years, POM-based materials have demonstrated remarkable potential in alkali metal-ion battery electrode design. For instance, one study employed polyoxovanadate (NH_4_)_2_[V^IV^_3_V^V^_3_O_10_(NH_2_C(CH_2_O)_3_)_3_] as building blocks to construct a 3D covalent organic framework (POF-1) via hydrothermal synthesis. This material features a non-interpenetrating diamond-type structure that effectively exposes active sites of monodispersed tris-V_6_O_19_ clusters, facilitating V^IV/V^ redox utilization and surface mass transport. As a lithium-ion battery cathode, POF-1 delivers a reversible capacity of 887.4 mAh·g^−1^ at 0.1 A·g^−1^ with >92% capacity retention after 1000 cycles (Figure 6a,b). Mechanistic studies reveal that both V centers and linker carbonyl groups serve as primary Li^+^ storage sites, accommodating up to 14 Li^+^ per unit.

Another study developed a MnPMo_8_V_6_@MXene/MoS_2_ ternary heterostructure through hydrothermal-electrostatic self-assembly. The Keggin-type POM and MoS_2_ were uniformly anchored on layered MXene, synergistically enhancing Faradaic activity, structural stability, and conductivity (Figure 6c). This composite electrode achieves 1061.3 mA·h·g^−1^ at 0.1 A·g^−1^, maintaining 851.1 mA·h·g^−1^ after 300 cycles, while retaining 450.3 mA·h·g^−1^ at 2 A·g^−1^, demonstrating exceptional rate capability and cycling stability.

To address sluggish ion diffusion kinetics in sodium-ion batteries, Wang et al. investigated Na^+^ occupation and migration in Na_3_V_2_(PO_4_)_3_ (NVP) via density functional theory (DFT)calculations. As shown in Figure 6d, the results indicate that Na (1) sites exhibit greater thermodynamic stability than Na (2) sites, rendering only Na (2) sites electrochemically active during cycling. Three possible diffusion pathways were analyzed, revealing that cooperative ion exchange involving both Na (1) and Na (2) sites is energetically favorable. Elemental doping at Na (1) sites effectively modulates the lattice parameters of NVP and discharge potential. Furthermore, a dual nitrogen-modified N-NVP/N-CN composite was designed, featuring surface N-doped NVP coupled with N-doped carbon nanocages. This architecture reduces Na^+^ diffusion barriers, enhances electron transport, mitigates stress, and provides abundant reaction interfaces through its porous structure, significantly improving electrochemical performance (Figure 6e). In recent years, polyoxometalate-based solid-state electrolytes have also demonstrated remarkable potential. For instance, the Li_3_PW_12_O_40_ electrolyte exhibits a high ionic conductivity of 0.89 mS·cm^−1^ and a low activation energy of 0.23 eV. Solid-state lithium batteries assembled with this electrolyte achieve stable cycling at a high voltage of 4.35 V with an areal capacity exceeding 4 mAh·cm^−2^, while the raw material cost is reduced to $5.68 per kilogram. Furthermore, the isomorphous substituted Li_3_PMo_12_O_40_ can form a low-resistance interface with tungsten-based electrolytes, enabling a long cycling life of 650 cycles in lithium-air batteries [128].

As for electrode design, sub-nanometer structures effectively reconcile the conflict between volume expansion and active site utilization. A sub-1 nm hybrid anode integrating phosphomolybdic acid clusters with transition metal oxides utilizes molecular-scale dispersion and electron-rich properties to enhance lithium-ion interfacial kinetics, achieving a high reversible capacity of 1157 mAh·g^−1^ at 100 mA·g^−1^. By employing polydopamine as a molecular bridge between PMo_12_ clusters and graphene, spatial confinement suppresses aggregation and dissolution, while nitrogen sites promote sodium-ion intercalation and the graphene framework ensures continuous electron conduction and mechanical support, enabling full electrochemical activity of all POM clusters with maintained electrode integrity [129].

POMs demonstrate potential as high-capacity electrodes or functional additives in lithium-ion batteries, yet their commercialization is constrained by three fundamental challenges. Scalable synthesis remains problematic, as POM preparation typically requires intricate hydrothermal methods and crystallization control, making it difficult to achieve ton-scale production while maintaining structural fidelity; additional steps such as nano-structuring and carbon compositing further complicate manufacturing. Although POMs are composed of earth-abundant elements like tungsten and molybdenum, their overall cost is elevated by expensive high-purity precursors and energy-intensive synthesis, rendering them less competitive than commercial cathode materials such as lithium iron phosphate or NMC. Most critically, long-term stability concerns persist: POM dissolution in organic electrolytes leads to active material loss and electrode degradation, while severe volume changes during cycling cause particle pulverization. Concurrently, unstable solid-electrolyte interphase formation coupled with multi-electron reactions accelerates capacity fading. Furthermore, the compatibility of POM frameworks with high-voltage conditions (>4 V) and their gas generation behavior remain insufficiently validated. Overcoming these barriers requires developing streamlined one-pot synthesis routes, constructing robust POM-carbon hybrid architectures, and systematically evaluating cycling durability across full voltage windows and wide temperature ranges.

#### 4.3.2. Lithium–Sulfur Batteries

Lithium–sulfur (Li-S) batteries are regarded as one of the most promising next-generation high-energy-density storage systems, owing to the abundance, low cost, and environmental friendliness of sulfur-based cathodes [134,135]. The operation mechanism relies on the reversible cleavage and formation of S-S bonds, enabling the conversion between chemical and electrical energy. During discharge, elemental sulfur is progressively reduced to soluble long-chain lithium polysulfides (Li_2_S_x_, 4 ≤ x ≤ 8), which are further converted into insoluble short-chain sulfides (Li_2_S_2_/Li_2_S). The charging process involves the reverse oxidation reactions, ultimately regenerating elemental sulfur [136,137]. Despite considerable application potential, the practical deployment of Li-S batteries faces several critical challenges:

(i) Polysulfide Shuttle Effect: Soluble long-chain polysulfides generated during cycling dissolve into the electrolyte, migrate through the separator, and undergo reduction at the lithium anode to form insoluble short-chain sulfides. This leads to active material loss, capacity fading, and diminished Coulombic efficiency. irreversible detachment of polysulfides from the conductive matrix further exacerbates capacity decay [138,139];

(ii) Poor Electrical Conductivity: Both elemental sulfur and its discharge products (Li_2_S_2_/Li_2_S) are electronically insulating, resulting in sluggish reaction kinetics. The deposition of short-chain sulfides on the electrode surface reduces effective triple-phase boundaries and triggers competition between polysulfide migration and interfacial deposition, severely limiting capacity utilization and rate capability [140];

(iii) Cathode Volume Expansion: The transformation from S_8_ to Li_2_S involves a substantial volume expansion (~80%), which can cause electrode structural degradation, active material detachment, and loss of electrical contact, leading to rapid capacity decay [141];

(iv) Li Anode Corrosion and Dendrite Growth: The lithium metal anode suffers from dual challenges of dendrite formation and polysulfide-induced corrosion. Localized current density inhomogeneity promotes Li dendrite growth, raising internal short-circuit risks. Concurrently, dissolved polysulfides participate in parasitic reactions at the Li surface, inhibiting the formation of a stable solid electrolyte interphase (SEI) and accelerating lithium corrosion and electrolyte consumption [142];

(v) SEI Instability: Polysulfide migration to the anode disrupts the native SEI layer, while repeated lithium plating/stripping causes mechanical deformation, resulting in continuous SEI fracture and reformation. This process consumes active lithium and electrolyte, reducing Coulombic efficiency and cycle life [143].

**Figure 7:**
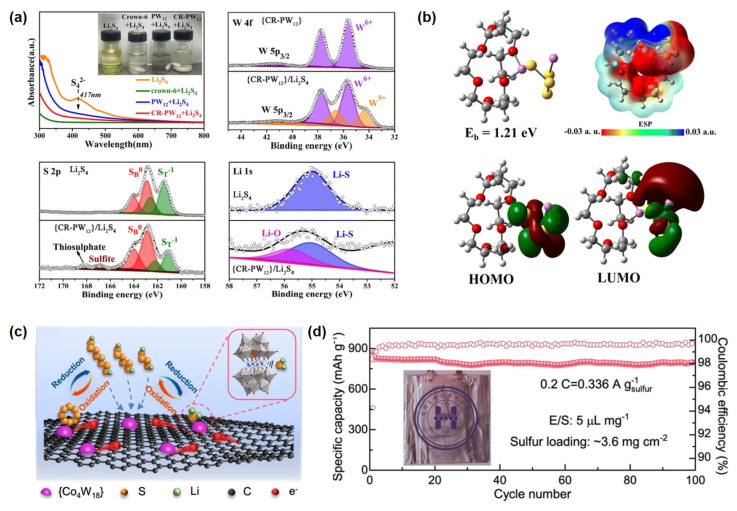
(**a**) The conversion mechanism of CR−PW_12_ toward LiPSs. (**b**) Theoretical calculations on the conversion of LiPSs by CR− PW_12_: electrostatic potential maps (ESPs) and frontier molecular orbital diagrams. Reprinted with permission from Ref. [145], Copyright 2023, *Chemical Communications*. (**c**) Schematic illustration of a bifunctional catalytic effect for Li_2_S deposition and oxidation. (**d**) Cycling performance of a pouch cell. Reprinted with permission from Ref. [77], Copyright 2022, *Chemical Communications*.

Recently, POM-based functional nanomaterials have been introduced into Li-S cathode design owing to their highly electronegative cluster structures and superior redox properties [26,144]. These materials can effectively anchor and catalytically convert polysulfides, suppressing the shuttle effect and significantly enhancing cycling stability and rate performance. For example, Yang et al. [145] successfully constructed a functional supramolecular material, denoted as CR-PW_12_, with the formula Li_3_Cl[(HPW_12_O_40_)(H_24_C_12_O_6_)_3_(CH_3_CN)_2_], by immobilizing a POM catalyst within a solid-state supramolecular hybrid framework. This material exhibits remarkable chemical stability, tunable topology, and unique reversible adaptivity, which not only enhance the structural stability of the POM catalyst within the hybrid matrix but also enable dynamic capture and release of LiPSs through supramolecular recognition, thereby significantly facilitating lithium-ion transport in Li-S batteries. Studies demonstrate that CR-PW_12_ exhibits strong chemisorption toward soluble LiPSs, effectively anchoring them. Moreover, leveraging the multi-valency of tungsten, it catalyzes the conversion of Li_2_S_4_ to higher-order Li_2_S_6_ and sulfur oxy-species (SO_x_) (Figure 7a). Theoretical calculations further reveal that CR-PW_12_ encapsulates Li_2_S_4_ and Li_2_S_6_ via host–guest interactions and Li-O bonding, forming inclusion complexes. Theoretical calculations results indicate that the HOMO and LUMO of these complexes are primarily localized around the polysulfide molecules, confirming their role as a supramolecular host for reversible polysulfide storage and release during cycling, thus effectively suppressing the shuttle effect (Figure 7b). Additionally, Zhang et al. synthesized a crystalline compound, (HAV)[PMo_12_O_40_]·6H_2_O (labeled as AV-PMo_12_), using Keggin-type phosphomolybdic acid (H_3_PMo_12_O_40_·3H_2_O) and aminopropyl viologen (AV) as precursors, connected through intermolecular hydrogen bonding and electrostatic interactions. This compound demonstrates bidirectional electrocatalytic activity during cycling: PMo_12_ undergoes reversible redox transitions between oxidized and reduced states, while AV facilitates electron capture and transfer to PMo_12_ via hydrogen bonds, enhancing its electron-accepting ability and consequently improving the catalytic conversion of LiPSs. Additionally, PMo_12_ promotes Li^+^ desolvation, accelerating ion transport and enabling uniform lithium deposition. Electrochemical tests show that Li-S cells with AV-PMo_12_-modified separators retain a reversible capacity of 469 mAh g^−1^ after 1000 cycles at 2.0 C, with a capacity decay rate of only 0.034% per cycle at 5.0 C over 1000 cycles. Furthermore, a composite material consisting of a POM framework ([Co_4_(PW_9_O_34_)_2_]^10−^) and multilayer reduced graphene oxide has been reported as a single-dispersed molecular cluster catalyst [77]. By leveraging interfacial charge transfer and exposed unsaturated cobalt sites, the composite exhibits efficient polysulfide adsorption and reduced energy barriers for polysulfide conversion, serving as a bifunctional electrocatalyst (Figure 7c). Under high sulfur loading of 5.6 mg cm^−2^ and low E/S ratio of 4.5 μL mg^−1^, the assembled cell delivers an areal capacity of 4.55 mA h cm^−2^. Moreover, a pouch cell configured with this catalyst maintains a specific capacity of approximately 800 mA h g^−1^ after 100 cycles at 0.2 C with a sulfur loading of 3.6 mg cm^−2^ and an E/S ratio of 5 μL mg^−1^ (Figure 7d).

In separator modification, Wu et al. developed a functional coating strategy using litigated polyoxometalate Li_3_PW_12_O_40_ [146]. This coating exhibits dual adsorption-catalysis functionality, maintaining a high Li^+^ transference number (0.84) while effectively suppressing polysulfide migration and accelerating reaction kinetics. Electrochemical performance testing shows that Li-S batteries with the modified separator achieve an initial discharge capacity of 1510.2 mAh g^−1^ at 0.5 C, retaining 710.9 mAh g^−1^ after 400 cycles. Even under harsh conditions-high sulfur loading (8.0 mg cm^−2^) and low E/S ratio (3.0 µL mg^−1^)-the pouch cell delivers a high energy density of 379 Wh kg^−1^.

POMs have demonstrated remarkable capabilities as electrocatalysts for polysulfide conversion in lithium–sulfur batteries, yet their commercial deployment faces critical hurdles. Scalable synthesis remains challenging due to the reliance on complex templating agents and precise pH control during hydrothermal reactions, hindering kilogram-scale production with consistent catalytic site integrity; further complications arise during porous substrate integration. Although POMs utilize low-cost metals (e.g., W and Mo), their overall cost is inflated by template consumption, energy-intensive synthesis, and noble-metal modification strategies (e.g., Ce/V doping), making them economically uncompetitive against conventional carbon hosts. The most pressing concern involves long-term stability: gradual dissolution in ether-based electrolytes diminishes active species and catalytic activity, while their strong indicability accelerates electrolyte decomposition and anode interface thickening. Under practical conditions—such as high sulfur loading and lean electrolytes—the synergistic anchoring-catalysis function of POMs degrades due to structural distortion, leading to rapid capacity fading. Addressing these limitations requires developing eco-friendly mass synthesis techniques, designing electrolyte-compatible encapsulation architectures, and rigorously validating cycle life under realistic operating parameters (e.g., high areal capacity and limited electrolyte).

#### 4.3.3. Aqueous Zinc-Ion Batteries

Aqueous zinc-ion batteries demonstrate promising potential for large-scale energy storage due to their high safety, low cost, and considerable energy density [147,148]. However, several critical challenges impede their practical application:

(i) Zn anodes suffer from dendrite growth, hydrogen evolution reaction, and corrosion, while byproduct accumulation impedes ion transport and increases interfacial resistance [149];

(ii) Cathode materials (e.g., Mn-/V-based compounds) exhibit structural dissolution and degradation during cycling, and the sluggish diffusion kinetics of Zn^2+^ limit rate capability [150,151];

(iii) The narrow voltage window (~1.23 V) and freezing tendency at low temperatures of aqueous electrolytes restrict energy density and operating temperature range [152,153].

In this regard, POMs offer a promising strategy to address these issues through their asymmetric surface charge distribution, strong interaction with Zn, and reversible redox activity, which collectively regulate electrolyte coordination, suppress side reactions, and provide additional electrode capacity.

**Figure 8:**
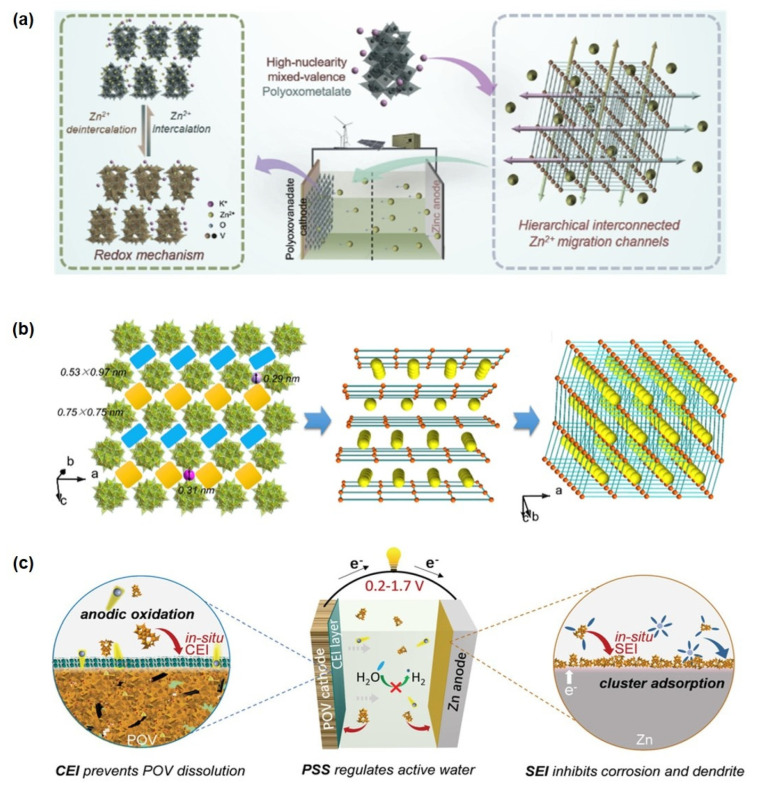
(**a**) Energy storage mechanism diagram of a high−nuclearity mixed-valence POM with hierarchically interconnected Zn^2+^ migration channels. (**b**) Topologic images of hierarchical interconnected channels. Reprinted with permission from Ref. [154], Copyright 2020, *Nano Energy*. (**c**) Multifunctionality illustrations of the POV cluster. Reprinted with permission from Ref. [155], Copyright 2022, *Advanced Energy Materials*.

Yang et al. [154] pioneered the application of nanoscale mixed-valence K_10_[V^IV^_16_V^V^_18_O_82_] (KVO) clusters in zinc-ion storage systems, constructing a Zn/HNPC battery with exceptional charge-discharge performance (Figure 8a). The material features multi-dimensional interconnected Zn^2+^ migration channels formed through 3D ordered stacking of nanoclusters, significantly enhancing electron conduction, Zn^2+^ diffusion rate, and migration efficiency (Figure 8b). Electrochemical tests demonstrated that the KVO-based battery delivers a high reversible capacity of 401 mAh g^−1^ at 0.1 A g^−1^, retains 93% capacity after 4000 cycles at 3 A g^−1^, and achieves an energy density of 285 Wh·kg^−1^ with a power density of 4.5 kW kg^−1^. Subsequently, Zhou et al. developed a potassium zinc vanadate (KZVO) cathode material via dehydration of KZVO. The assembled Zn-ion battery exhibited a discharge capacity of 223.4 mAh g^−1^ at 0.1 A g^−1^ with 97.6% capacity retention after 50 cycles. At a high current density of 2 A g^−1^, the system maintained nearly 100% capacity retention (145 mAh g^−1^) after 800 cycles, along with an energy density of 182.9 Wh·kg^−1^, power density of 40.38 W kg^−1^, and a Zn^2+^ diffusion coefficient on the order of 10^−10^ cm^2^ s^−1^. In the hierarchically structured L-rGO/PPy@POM composite constructed via a redox-relay Heck reaction, in situ and ex situ spectroscopic analyses confirm that the Mo=O bond participates more significantly in Zn^2+^ insertion compared to the two Mo-O single bonds and the edge-/corner-sharing Mo-Mo bonds. As a result, this material maintains a capacity of 68.0 mAh g^−1^ even at an ultrahigh current density of 10,000 mA g^−1^, with 80.4% capacity retention after 22,000 cycles. Furthermore, the corresponding flexible full cell achieves a volumetric energy density of 51.1 mWh cm^−3^ [37].

Further extending functional design, Yang et al. [155] proposed a triple-functional strategy centered on the K_10_[V^IV^_16_V^V^_18_O_82_] (POV) cluster, simultaneously enhancing cathode structural integrity, electrolyte compatibility, and Zn anode reversibility. Their results indicate that an anodically induced interphase effectively suppresses POV cathode dissolution. Theoretical simulations revealed that the [V^IV^_16_V^V^_18_O_82_]^10−^ anion can reconstruct the primary solvation sheath of Zn^2+^, mitigate side reactions, and facilitate uniform zinc nucleation by forming a Zn-POV solid-state interphase, thereby inhibiting dendrite growth (Figure 8c). In the realm of interface regulation and electrolyte engineering, polyoxometalates (POMs) demonstrate unique “ion siphon” and interface reconstruction functionalities [156]. Studies show that constructing a redox-active solid electrolyte interphase (SEI) by adding 2 mM K_6_V_10_O_28_·9H_2_O effectively neutralizes the negative effects of the space charge layer. This enables symmetrical Zn cells to achieve stable cycling for over 1200 h at 10 mA cm^−2^, while the assembled Zn-ion hybrid capacitors maintain 92.4% capacity retention after 80,000 cycles. Beyond vanadium-based systems, a water-soluble and highly stable high-nuclearity (Nd_9_Si_4_W_39_) polyoxotungstate cluster has been reported [157]. Upon interaction with Zn^2+^, this discrete cluster undergoes structural reorganization into an extended 2D honeycomb-like framework ([Zn(H_2_O)_4_]_3_[Nd_9_Si_4_W_39_]_2_), demonstrating atomistic Zn^2+^-mediated reconstruction. Notably, this transformation enables its use as an efficient electrolyte additive, inducing a protective interfacial layer on the Zn metal surface that significantly improves electrode reversibility. Symmetric Zn//Zn cells with this additive exhibited a cycling lifespan exceeding 2000 h at 1 mA cm^−2^.

POMs demonstrate promise as high-performance cathode materials for aqueous zinc-ion batteries, yet their commercialization faces three primary challenges. Scalable synthesis remains difficult due to the sensitivity of POM crystallization to precise temperature and pH control, resulting in batch-to-batch inconsistencies; nano-engineering processes such as carbon coating or 3D framework construction further complicate mass production. Although POMs utilize earth-abundant elements like Mo and V, cost volatility of high-purity precursors (particularly vanadates) and energy-intensive purification make them less competitive than alternatives such as Prussian blue analogues. Most critically, operational stability concerns persist: Zn^2+^ insertion often induces phase transitions and structural distortion in POM frameworks, leading to rapid capacity fade, while chemical dissolution in aqueous electrolytes causes active material loss. Additional systemic issues, including hydrogen evolution reaction and interfacial instability, require further resolution. Future advances should focus on developing continuous-flow synthesis, designing composite electrodes with buffering architectures, and evaluating cycling durability under practical conditions (e.g., high areal capacity and wide temperature range).

## 5. Conclusions and Outlook

POMs demonstrate unique advantages in electrochemical energy storage due to their reversible multi-electron transfer capabilities and tunable molecular structures (Table 1). This review systematically elucidates three key functional mechanisms of POMs in energy storage systems: First, as molecular-scale redox mediators, their distinctive framework structures effectively buffer volume strain during ion insertion/extraction, significantly enhancing the cycling stability of lithium/sodium-ion batteries. Second, through Lewis acid–base dual-functional sites, they form strong chemical interactions with polysulfides, fundamentally suppressing the shuttle effect in lithium–sulfur batteries. Third, their fast and reversible surface redox reactions provide additional pseudocapacitive contributions to hybrid supercapacitors, effectively compensating for the capacity limitations of conventional electrode materials.

Despite remarkable laboratory achievements, the industrialization of POMs faces three major challenges. Regarding intrinsic material properties, the structural evolution of POM clusters during continuous charge/discharge cycles remains unclear, particularly lacking direct experimental evidence for the reversible breakage/formation mechanism of metal–oxygen bonds. In synthesis methodology, conventional hydrothermal methods struggle to maintain molecular-level monodisperses at scale, while aggregation during electrode preparation severely reduces active site utilization. For device integration, high interfacial energy barriers with commercial current collectors lead to sluggish charge transfer kinetics, and dissolution in organic electrolytes remains unresolved. To overcome these bottlenecks, future research requires multidisciplinary strategies. Mechanistically, time-resolved in situ techniques should be developed to track coordination environment evolution during electron transfer processes. Material-wise, vanadium-based POMs with open frameworks and lanthanide-doped hetero-poly acids show promising electron spin modulation, though their structure–property relationships remain underexplored. Practically, standardized evaluation protocols and surface modification strategies are urgently needed to enhance compatibility with existing battery manufacturing.

Future investigations should prioritize the following: (1) integrating artificial intelligence with theoretical calculations to establish structure–property relationships; (2) employing advanced in situ characterization to decipher phase evolution and side reactions; and (3) developing novel POM materials through molecular engineering. These efforts will establish a complete technology chain from molecular design to device integration. By overcoming critical challenges in material stability, process compatibility, and interface engineering, POM-based electrochemical energy storage technologies will provide innovative solutions for next-generation energy storage systems, ultimately advancing practical sustainable energy technologies.

## Figures and Tables

**Table 1:** POM-based materials for batteries and supercapacitors.

Materials	Capacity (mAh g^−1^)	Current/Current Density/Rate	Capacity Retention	Power Density	Energy Density	Cycle Number	Ref.
Li_3_PW_12_O_40_	165.6	0.1 C	91.5%			200	[128]
L-rGO/PPy@POM	60	10 A·g^−1^	80.4%	6.2 mW·cm^−3^	29.3 mWh·cm^−3^	22,000	[36]
BAS-MOF-1	708.2	0.1 A·g^−1^	94%			300	[158]
MZC-PMA HSNSs	592	1 A·g^−1^	100%			700	[159]
PMo_12_/PDA@rGO	277.5	1 A·g^−1^	99.89%			2000	[129]
LNLPW	1510.2	0.5 C	40%		379 Wh·kg^−1^	400	[146]
NVO	814	0.1 C	70.5%			200	[61]
POMs-D@CNFs	170	3 A·g^−1^	100%	16,000 W·kg^−1^	30.2 Wh·kg^−1^	8000	[120]
POM-based Ag-MCFs	770	0.1 A·g^−1^	61%			100	[160]
Li_7_[V_15_O_36_(CO_3_)]	173	2 A·g^−1^	60%	25.7 kW·kg^−1^	300 Wh·kg^−1^	100	[25]
NAM-EDAG	1000	0.1 A·g^−1^	100%			100	[23]
SWCNTs-OH-P-PMo_12_-400	10.2 mF·cm^−2^	1 mA·cm^−2^	99.3%	7 μW·cm^−2^	0.71 μWh·cm^−2^	1000	[161]
EMI-Mo_72_V_30_@rGO	1150	0.1 A·g^−1^	100%			100	[162]
CuMo_16_	194.19	1 A·g^−1^	68.2%	500 W·kg^−1^	97.1 Wh·kg^−1^	2500	[163]
MoSe_2_/(Ni, Co)Se_2_	359.9	1 A·g^−1^	83.7%	800.9 W·kg^−1^	40.3 W h·kg^−1^	10,000	[121]
POM/Fe_2_O_3_/PANI	528 F·g^−1^	0.2 A·g^−1^	91.62%	7.14 kW kg^−1^	73.4 Wh·kg^−1^	100	[164]
NiMoO_4_·MoO_2_/NC	364.96 F·g^−1^	1 A·g^−1^	80%	4140 W·kg^−1^	102 Wh·kg^−1^	10,000	[122]
6CDs@PMo_12_/Ni-MOF	155.8 F·g^−1^	5 A·g^−1^	98.4%	749.9 W·kg^−1^	39.22 Wh·kg^−1^	20,000	[123]
POMs-ILs@MOFs	600	1 A·g^−1^	99.98%			400	[165]

## Data Availability

No new data were created or analyzed in this study.
